# Trigeminal Neuralgia Induced Headache: A Case Report and Literature Review

**DOI:** 10.7759/cureus.9226

**Published:** 2020-07-16

**Authors:** Saurabh Kataria, Zahoor Ahmed, Unaiza Ali, Sarfaraz Ahmad, Anum Awais

**Affiliations:** 1 Neurology, University of Missouri, Columbia, USA; 2 Internal Medicine, King Edward Medical University, Mayo Hospital, Lahore, PAK; 3 Internal Medicine, Ziauddin University, Karachi, PAK; 4 Internal Medicine, Saint James School of Medicine, Chicago, USA; 5 Internal Medicine, Fatima Jinnah Medical University, Sir Ganga Ram Hospital, Lahore, PAK

**Keywords:** headache, migraine, trigeminal neuralgia, carbamazepine

## Abstract

A 51-year-old woman with a past medical history of migraine presented with severe headache for the last three weeks. The pain was intermittent and throbbing in nature. She has not experienced any headaches in the past several years. She took her migraine pills and over-the-counter analgesics, but the pain did not resolve. Initial evaluation including physical exam and neurological exam was normal. Her serum chemistry was unremarkable and CT scan of the brain was nonsignificant. The patient was diagnosed with possible trigeminal neuralgia and the pain resolved after being started on tegral (carbamazepine). Unilateral headache is a typical presentation of atypical trigeminal neuralgia and is rarely reported in literature.

## Introduction

Trigeminal neuralgia is also known as tic douloureux; it is a common facial pain disorder. The pain typically involves the lower face and jaw, although sometimes it affects the area around the nose and above the eye [1]. This severe shooting pain is usually caused by the irritation of the fifth cranial nerve, which has its innervation to the forehead, cheek, and lower jaw and usually involves one side of the face [2]. The signs and symptoms of many pain conditions square measure just like those of trigeminal neuralgia. However, headache is uncommon in trigeminal neuralgia. Here we present a rare case of trigeminal neuralgia with a severe headache.

## Case presentation

A 51-year-old woman with a past medical history of migraine presented in the clinic with complaints of severe and throbbing pain on the right side of her head. The pain was episodic and lasted for 12-18 hours followed by a short period of relief. The pain had no association with aura, and the patient was symptom-free between headache. She was having this pain for the last three weeks. She had a history of migraine headaches since her adulthood. She had not experienced any headaches in the past several years. In the past, she was prescribed sumatriptan and naproxen sodium to treat migraine, but she had not needed them for years. After a few weeks of her new headache, her doctor prescribed migraine pills, which she took over the course of three days, but she did not get any kind of relief. The patient was overweight. Otherwise, she was in good health with no obvious problems.

The initial evaluation showed a temperature of 37°C, blood pressure of 120/70 mmHg, heart rate of 91 beats per minute, respiratory rate of 20/minute, and oxygen saturation of 99% on room air. On physical examination, the patient appeared healthy, alert, and oriented with intact cognition. Her skin, extremities, and pulses were normal, and her abdominal examination was normal. However, she appeared to be in pain and the area of head involving pain is highly sensitive to light touch. On her neurological examination, her power, coordination, and gait were intact, and her toes were down going. The sensation was intact bilaterally, and the reflexes were also intact on both sides of the body. Her cranial nerve examination was also normal. There was no evident deformity on her face or eye, and she did not have any facial muscle weakness. MRI revealed no abnormality.

The initial laboratory analysis is shown in Table 1. The patient’s platelet count, hemoglobin, hematocrit, and white blood cells were within normal ranges. Her serum chemistry was unremarkable.

**Table 1:** Results of hematological examination and metabolic panel. WBC, white blood cells; RBC, red blood cells; AST, aspartate aminotransferase; ALT, alanine aminotransferase

Parameter	Lab value	Reference
WBC count, cells/mm^3^	6000	4000–10,000
RBC count, million cells/mm^3^	4.3	4.2 to 5.4
Hemoglobin, g/dL	14.2	14–17
Hematocrit, %	42.8	41–51
Platelet count/mm^3^	179,000	150,000–350,000
Sodium, mmol/L	138	136–145
Potassium, mmol/L	3.9	3.5–5.0
Chloride, mmol/L	100	98–106
Urea nitrogen, mg/dL	13	8–20
Creatinine, mg/dL	0.9	0.7–1.2
Blood glucose, mg/dL	101	70–100 (Fasting)
Total bilirubin, mg/dL	0.08	0.3–1.2
AST, IU/L	31	5–40
ALT, IU/L	42	7–56

The patient was treated with tegral (carbamazepine) at a dose of 200 mg twice per day with a possible diagnosis of trigeminal neuralgia after ruling out other possible causes carefully. Her symptoms improved gradually, but she experienced severe drowsiness and nausea as the side effects of carbamazepine. After a few days of treatment, the pain resolved, and she quit taking medication. Four weeks later, the patient presented again with the same signs and symptoms. Her pain again subsided within two weeks with the same dose (200 mg x 2/day) of carbamazepine, but she was too drowsy. She experienced a similar reaction when she was switched to lamotrigine, which also improved symptoms but also resulted in excessive sleepiness. She continued on carbamazepine for five weeks further, and her symptoms eventually resolved. At her most recent follow-up six months after the recurrence, she had not had any more symptoms.

## Discussion

Trigeminal neuralgia is a localized peripheral neuropathy caused by the irritation of the fifth cranial nerve (the trigeminal nerve). It is characterized by severe intermittent facial pain, which usually involves one side of the face. The pain is typical of a throbbing nature [3]. It is also called tic douloureux (painful tic) because patients often distort the face because of severe pain. This patient has an atypical presentation of trigeminal neuralgia. She had pain that is limited to her head, not her face, which is a typical presentation of atypical neuralgia [4]. The head pain rather than pain around the face is usually caused by several factors. She may have had an unusual pain pattern due to an unusual type of trigeminal neuralgia. Head and scalp hypersensitivity because of migraine headaches can also result in the pain. As the patient has a history of migraine headaches that may have involved her face, pain due to trigeminal neuralgia may have been similar to that of migraines.

Migraine headaches usually cause facial pain, occasionally without a headache at all. As our patient has a history of migraine headaches lasting six to 12 hours for many years, her migraine headache was associated with aura, usually preceded by nausea. It is also possible that the explanation and presentation of her signs and symptoms could have been the outcome of her own bias. This patient has unilateral pain, and this unilateral symptom is not a typical feature of migraine headaches. The facial hypersensitivity upon physical examination and her improvement of the symptoms with carbamazepine rather than migraine pills were clues that advocated trigeminal neuralgia.

Trigeminal neuralgia affects males and females of all ages. Trigeminal neuralgia has a prevalence of 0.1 to 0.2 per one thousand and an incidence ranging from four to 20 cases per 100,000 people per year. The female to male ratio is around 3:2 [5]. While it is common after 50 years of age, trigeminal neuralgia is uncommon in young adults and rare in children [6]. The right side of the face is reported being more commonly involved [7].

Trigeminal neuralgia is usually idiopathic. However, any factor irritating the trigeminal nerve can cause it. Inflammation and compression of the trigeminal nerve are the common risk factors associated with trigeminal neuralgia. Inflammation is usually caused by an infection, and compression of trigeminal results from the blood vessel or tumor. Trigeminal neuralgia also has an association with multiple sclerosis and inflammatory or autoimmune conditions [8]. Any trigger such as a light stimulus to the region supplied by the trigeminal nerve may lead to the onset of symptoms. An increased risk of trigeminal neuralgia in migraine patients has been recently recognized [9]. The association between migraine and trigeminal neuralgia remained significant in sensitivity analyses. Among migraine subtypes, patients with migraine and aura were at greater risk of trigeminal neuralgia development [9]. The condition is not dangerous, but severe persistent pain can be limit daily activities. Anticonvulsant carbamazepine or oxcarbazepine is the drug of choice for the patients with trigeminal neuralgia, and most of the patients respond well [10]. Decompression of the blood vessels might cure the pain in patients with possible anatomical etiology for trigeminal neuralgia.. A trigeminal ganglion nerve block or injections with botulinum toxin may be considered if there is no anatomical etiology identified [11]. Rhizotomy or gamma knife surgery to severe the nerve root may also be considered for refractory and treatment-resistant trigeminal neuralgia. However, these procedures can result in impairment of facial sensation [12].

## Conclusions

It is important to consider other causes of pain apart from migraine headaches in patients who have a history of migraine headaches. Atypical trigeminal neuralgia presents with involvement of head, rather than the face that is more typical of the condition. Trigeminal neuralgia resolves with anticonvulsant carbamazepine if taken properly.
